# Shape and secondary structure prediction for ncRNAs including pseudoknots based on linear SVM

**DOI:** 10.1186/1471-2105-14-S2-S1

**Published:** 2013-01-21

**Authors:** Rujira Achawanantakun, Yanni Sun

**Affiliations:** 1 Department of Computer Science and Engineering, Michigan State University, Michigan, USA

## Abstract

**Background:**

Accurate secondary structure prediction provides important information to undefirstafinding the tertiary structures and thus the functions of ncRNAs. However, the accuracy of the native structure derivation of ncRNAs is still not satisfactory, especially on sequences containing pseudoknots. It is recently shown that using the abstract shapes, which retain adjacency and nesting of structural features but disregard the length details of helix and loop regions, can improve the performance of structure prediction. In this work, we use SVM-based feature selection to derive the consensus abstract shape of homologous ncRNAs and apply the predicted shape to structure prediction including pseudoknots.

**Results:**

Our approach was applied to predict shapes and secondary structures on hundreds of ncRNA data sets with and without psuedoknots. The experimental results show that we can achieve 18% higher accuracy in shape prediction than the state-of-the-art consensus shape prediction tools. Using predicted shapes in structure prediction allows us to achieve approximate 29% higher sensitivity and 10% higher positive predictive value than other pseudoknot prediction tools.

**Conclusions:**

Extensive analysis of RNA properties based on SVM allows us to identify important properties of sequences and structures related to their shapes. The combination of mass data analysis and SVM-based feature selection makes our approach a promising method for shape and structure prediction. The implemented tools, Knot Shape and Knot Structure are open source software and can be downloaded at: http://www.cse.msu.edu/~achawana/KnotShape.

## Background

Noncoding RNAs (ncRNAs), which are transcribed but not translated into proteins, play diverse and important biological functions in all living organisms [1]. Many types of ncRNAs perform their functions through both their sequences and secondary structures, which are defined by the interacting base pairs. Of the characterized secondary structures of ncRNAs, Watson-Crick (C-G and A-U) and wobble base p airs (G-U) are most commonly seen. As knowing the secondary structure provides important information to undefirstafinding the tertiary structures and thus the functions of ncRNAs, deriving the secondary structures of ncRNAs remains an important research topic in RNA informatics.

Pseudoknot is an important structural motif in secondary structures of many types of ncRNAs. Formally, a pseudoknot occurs when an RNA has two base pairs, *i *- *j *and *i' *- *j'*, such that *i *<*i' *<*j *<*j'*. Psuedoknots are known to play important functions in telomerase RNA, tmRNA, rRNA, some riboswitch, some protein-biding RNA, Viral ribosomal frameshifting signals, etc [2]. There are 26,704 sequences in 71 ncRNA seed families of Rfam 10.0 [3] containing pseudoknots. With advances in sequencing technologies and structure prediction, more pseudoknot structures are expected to be disclosed.

Many computational methods have been used to determine the native structure of ncRNAs. A native structure is a structure that forms conformationally folding in native state before forming the tertiary structure. The gap between the free energy of the native state and other non-native structures is often small [4]. Thus, misfolded conformations can form with high probabilities [5]. For a review of available tools, please see [6,7].

Although there is promising progress, finding the native secondary structure is still difficult. In particular, identifying the pseudoknot, an important structural motif in many types of ncRNAs, poses a great challenge for existing methods. Predicting the minimum free energy secondary structure that includes pseudoknots has been proven to be NP-hard [8]. One recent attempt is to first predict the abstract shapes (or shapes for short), which retain adjacency and nesting of structural features but disregard the length details of helix and loop regions [9]. The predicted shape will then be used to guide structure prediction. The idea of abstract shapes has long been used to characterize different types of structures. For example, most tRNAs have the clover-leaf structure; most pre-miRNAs have the stem-loop structure; many types of pseudoknots have an H-type structure.

While the size of the folding space of an RNA sequence increases exponentially with the sequence length [10], many possible folding only differ in the details of the loop and helix regions and hence have the same abstract shape. Previous analysis shows that the space of the abstract shapes is significantly smaller than the complete folding space [11]. Knowing the abstract shape can significantly reduce the search space for structure prediction tools and improves the accuracy of structure prediction [9,12]. The utilities of abstract shapes have been demonstrated in a number of recent publications. The Giegerich group used abstract shapes in comparative structure prediction in pseudoknot-free sequences [12]. People use shapes to aid miRNA pre-cursor prediction in large-scale studies [13,14]. Furthermore, shapes are used to index fast-expafinding ncRNA families in Rfam [3] and lead to efficient known ncRNA search [15].

Previous work focused on shape derivation and usage for pseudoknot-free ncRNAs. There is a lack of studies of the usage of shapes in pseudoknot structure prediction. In this work, we predict the consensus shape of a group of homologous ncRNAs that may contain pseudoknots. In addition, we develop a program that uses the consensus shape for consensus pseudoknot structure prediction. A majority of existing pseudoknot structure prediction tools often have topology restrictions such as H-type, recursive H-type [16-19], kissing hairpin, or complexity levels of pseudoknot using genus numbers [20]. Therefore, using the predicted abstract shapes of input sequences can help remove the topology restriction and leads to more general and practical pseudoknot structure prediction tools. Compared with existing tools, our tool has the following properties:

• While most existing shape prediction tools use a single sequence as input, we conduct comparative shape prediction on homologous ncRNAs that might contain pseudoknots. Experiments show that comparative structure or shape prediction, which derives the consensus structure or shape from a group of homologous sequences, can achieve better accuracy than using a single sequence [6,12,21].

• We can predict the abstract shapes of both pseudoknot-free and pseudoknot-containing sequences.

• Current tools use the shape probability [22] or the sum of energies of structures to rank shapes. We use multiple features by combining well-studied feature ranking methods and the support vector machine (SVM) method.

• We demonstrate the usage of the shape by applying it to pseudoknot structure prediction. The whole software package can be directly used to derive the consensus secondary structure of homologous ncRNAs. The consensus shape prediction tool named KnotShape and the corresponding consensus pseudoknot prediction tool named KnotStructure are publicly available at our website.

We tested our software on hundreds of RNA sequence sets. The experimental results show that we can achieve 18% higher accuracy than the state-of-the-art consensus shape prediction tools on pseudoknot free sequences. For pseudoknot-containing sequences, we achieve approximate 29% higher sensitivity and 10% higher positive predictive value in structure prediction than similar tools.

### Related work

Computational structure prediction can be divided into de novo structure prediction and comparative structure prediction, which derive structures from a single sequence and multiple homologous ncRNAs respectively. As our method is to derive the consensus shape and structure of homologous ncRNAs, we briefly introduce related work in comparative ncRNA structure derivation. There are three general approaches for structure derivation from multiple sequences: simultaneously align and fold, align-then-fold, and fold-then-align. It is computationally expensive to simultaneously align and fold pseudoknot structures. The performance of the align-then-fold pseudoknot prediction heavily depends on the quality of the alignment. Usually multiple sequence alignment (MSA) tools such as ClustalW [23] are used to generate the alignment as the input to the folding tool. However, common structures can be missed due to misalignment between sequences lacking significant similarity [24]. In this work, we design a pseudoknot prediction tool using the fold-then-align strategy that does not require an alignment as input. Tools based on fold-then-align use a de novo folding tool to construct a small but representative sample of the folding space, which consists of the predicted optimal and sub-optimal structures. Structures from the folding space are chosen to maximize the structural and sequence similarity.

A number of software packages exist to predict the abstract shape for a single sequence. The sum of energies or the accumulated Boltzmann probabilities of all structures within a shape have been used as main features for shape prediction. The latter is often referred to as the shape probability. Usually the shapes with small sum of energies or high shape probabilities are more likely to be the correct shapes. It is claimed in RapidShapes [22] that using shape probabilities has superior performance over free energy-based approach because of its independence on sequence length and base composition. However, exact computation of the shape probability incurs exponential computational cost to the sequence length [22]. Thus, various heuristics or restrictions [25,26] have been adopted for fast shape probability computation.

RNAcast [12] derives the consensus shape from homologous pseudoknot-free sequences based on the fold-then-align strategy. Structures are grouped based on their shapes and shapes are ranked by sum of free energies of structures within the shape in ascending order. The first-ranked shape is presented as the consensus shape. The consensus structure is derived from the lowest free energy structures of each sequence within the shape.

## Methods

### RNA structures and their representations

#### RNA structures and pseudoknots

**Figure 1:**
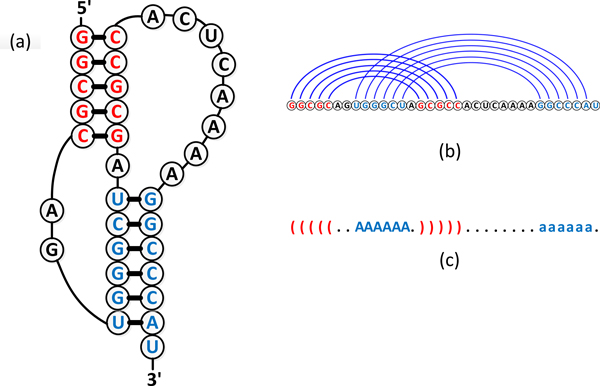
**Structure of an RNA pseudoknot**. (a-c) show the secondary structure, arc-based representation, and dot-bracket notation of mouse mammary tumor virus (MMTV) H-type pseudoknot with PDB code 1RNK. The bases in stacking regions are colored with blue while the unpaired bases are colored with black.

RNA molecules fold into complex three dimensional structures by nitrogenous bases that are connected via hydrogen bonds. The secondary structure of an ncRNA is defined by the interacting base pairs. Some RNA molecules fold into pseudoknot structures by paring bases in loop regions with bases outside the stem loop (Figure 1a).

In this work, two types of ncRNA secondary structure representations are used. The first type is the arc-based representation, where nucleotides and hydrogen bonds are represented by vertices and arcs, respectively (Figure 1b). For pseudoknot-free secondary structures, all arcs are either nested or in parallel. Crossover arcs indicate pseudoknots. The second type is based on dot-bracket notation, where '.' represents unpaired bases and matching parenthesis '(' and ')' indicate base-pairing nucleotides. Following the annotation of Rfam [3], we use an extended dot-bracket notation to represent pseudoknot structures. The base-pairing nucleotides forming pseudoknots are represented by upper-lower case character pairs, such as A..a or B..b, as shown in Figure 1c.

#### Abstract shapes

Abstract shapes were formally introduced by Giegerich et al. [9]. The folding space of a given RNA sequence is partitioned into different classes of structures, by means of abstracting from structural details. These classes are called abstract shapes, or shapes for short.

An RNA secondary structure can be mapped to an abstract shape with different levels of abstraction [12]. In the abstract shape, details about the lengths of the loop and stacking regions are removed (see Figure 1 for examples of stacking and loop regions). Stacking regions are represented by pairs of brackets and unpaired regions are represented by underscores.

**Figure 2:**
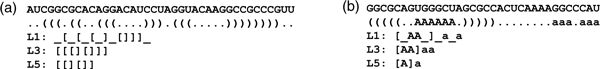
**Examples of abstract shapes in level 1, 3 and 5**. (a) The abstract shapes of a pseudoknot-free structure. (b) The abstract shapes of a structure with a pseudoknot.

Pseudoknots are represented by pairs of upper-lower case characters. Figure 2 presents examples of the abstract shapes of level 1, 3, and 5 of a pseudoknot-free structure and a pseudoknot. Level 5 represents the strongest abstraction and ignores all bulges, internal loops, and single-stranded regions. Level 3 adds the helix interruptions caused by bulges or internal loops. Level 1 only abstracts from loop and stack lengths while retains all single-stranded regions.

### Shape prediction

In this section we describe KnotShape, a comparative shape prediction tool for homologous ncRNA sequences that allows pseudoknots. The task of shape prediction is to select the best representative shape for given homologous sequences. In order to identify the best shape, various features such as shape probability [22], sum of energies of all structures in this shape [12], and the rank sum score [12] are evaluated to rank shapes. It has not been systematically assessed whether combinations of multiple features can lead to better shape prediction. In this work, we incorporate Support Vector Machine (SVM) [27] and feature selection techniques to determine important features for shape identification. In addition, we adopted a machine learning-based scoring function to evaluate the qualities of shapes.

The method contains two important components. The first one is the consensus shape prediction (Knot-Shape) and the second one is structure prediction using predicted shape as input (KnotStructure). We will first describe KnotShape, focusing on the feature construction and selection strategy. Then we will describe how to derive the consensus structure given the consensus shape.

#### Notation

**Figure 3:**
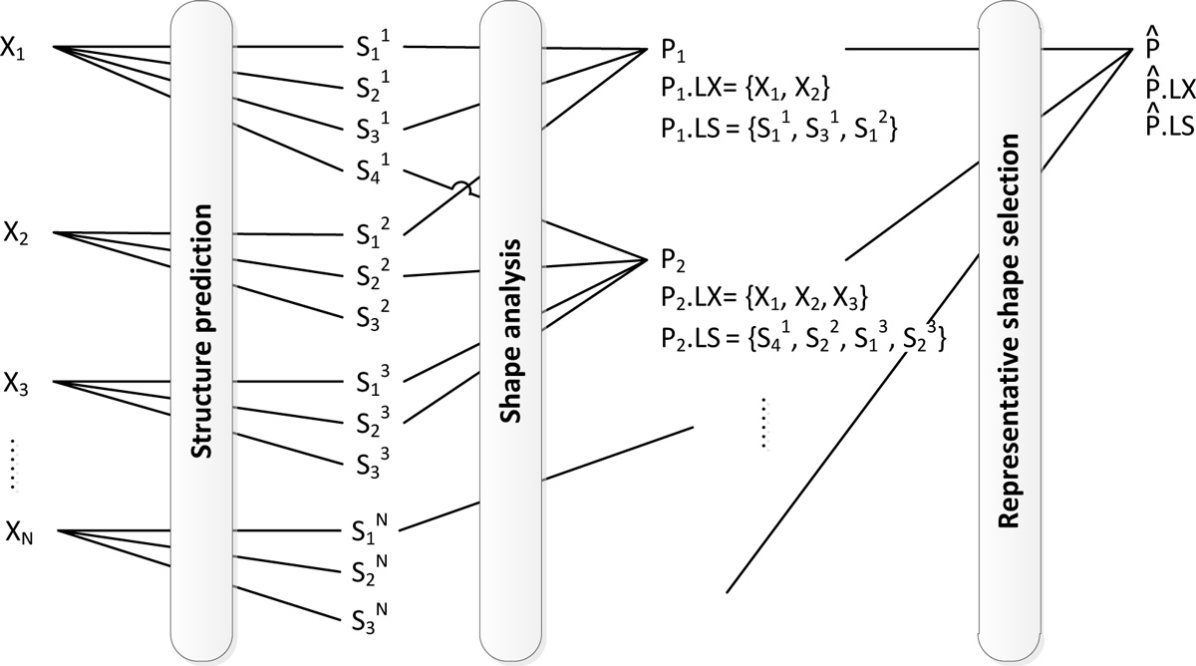
**The relationship between sequences, structures, and shapes**.

Figure 3 illustrates the mapping between sequences, structures, and shapes. The input is a set of homologous ncRNAs and the output is the predicted consensus shape. Notations used in this paper correspond to this mapping.

• The *N *homologous ncRNAs constitute the input sequence space. *X_i _*represents the *i*th sequence.

• Each sequence can be folded into different secondary structures. Let *S^i ^*represent the set of folded structures of the *i*th sequence *X_i_*. The set of structures predicted from all *N *input sequences is the union of *S^i^*: *S *= *S*^1 ^∪ *S*^2 ^∪ . . . ∪ *S^N^*.

• $S_{j}^{i}$ is the *j*th structure in the folding space of *X_i_*. Its free energy is denoted by $\Delta G\left(S_{j}^{i}\right)$. For a sequence *X_i_*, the minimum free energy MFE(*X_i_*) is the lowest free energy among the energies of all predicted structures of *X_i_*, i.e. $\text{MFE}(X_{i})=\min_{1\leq j\leq |S^{i}|}\Delta G(S_{j}^{i})$.

• All structures in *S *can be classified into a set of abstract shapes. For a shape *P*, we record its associated sequences and structures. *P.LX *denotes the set of associated sequences, each of which can fold into a structure with shape *P*. *P.LS *denotes all structures with shape *P*.

• $\hat{P}$ is the predicted shape of the given homologous sequences *X*_1_, *X*_2_, .., *X_N_*.

In order to explore the large folding space of multiple homologous sequences, we use a de novo folding tool to output the optimal and sub-optimal structures within a given energy cutoff. This heuristic does not allow us to explore the complete folding space. Given the observation that the correct structure is usually close to the optimal structure, this heuristic works well in practice [28].

#### Feature construction and selection

Intuitively, the correct shape tends to possess the following properties. The correct shape should have high shape probability, meaning that a large number of structures can be classified into this shape. When we have multiple homologous sequences as input, the correct shape should be well-represented by all or a majority of the input sequences. Also, the ranking of the structure with the correct shape in the folding space of each sequence should be high. In addition, some structures with the correct shape have low thermodynamic energies. For the energy-related properties, various measurements can be introduced. For example, instead of using the sum of the energies of all structures within a shape, one can use the smallest energy. Furthermore, more complicated properties such as the sequence similarity for all sequences associated with a shape *P *and the structural similarity of structures associated with a shape *P *might contribute to the shape prediction too. These similarities can be quantified using different methods such as k-mers profiles, multiple sequence alignment scores, variation of base pairs and so on.

It is not trivial to decide whether a single property is enough to choose the correct shape. If not, which combination of these properties can lead to the best shape prediction performance? In order to systematically choose a set of features (i.e. properties) for shape prediction, we use F-score [29] to measure the discrimination between a feature and its label. Given the feature vector *x_k_, k *= 1, .., *m*, the F-score of the *i*th feature is defined as:

$$F\left(i\right)\equiv \frac{{\left({\bar{x}}_{i}^{\left(+\right)}-{\bar{x}}_{i}\right)}^{2}+{\left({\bar{x}}_{i}^{\left(-\right)}-{\bar{x}}_{i}\right)}^{2}}{\frac{1}{n_{+}-1}\sum_{i=1}^{n_{+}}{\left({\bar{x}}_{k,i}^{\left(+\right)}-{\bar{x}}_{i}^{\left(+\right)}\right)}^{2}+\frac{1}{n_{-}-1}\sum_{i=1}^{n_{-}}{\left({\bar{x}}_{k,i}^{\left(-\right)}-{\bar{x}}_{i}^{\left(-\right)}\right)}^{2}}$$

where *n*_+ _and *n*_- _are the numbers of positive and negative instances respectively. ${\bar{x}}_{i}$, ${\bar{x}}_{i}^{\left(+\right)}$, and ${\bar{x}}_{i}^{\left(-\right)}$ are the average values of the *i*th feature of the whole, positive labeled, and negative labeled data. $x_{k,i}^{+}$ and $x_{k,i}^{-}$ are the values of *i*th feature of the *k*th positive and negative instances respectively.

F-score reflects the discrimination of the features. The higher the F-score, the more discriminative the feature is. F-score is known to have a disadvantage in that it does not carry out the mutual information between features as it considers each feature separately. However, F-score is simple and quite effective in practice.

Feature selection searches for the optimal subset of features [30]. There exist different methods for feature selection. In this work, we adopt sequential forward search (SFS) [31] because of its simplicity and effectiveness. Starting with an empty set, we iteratively select one feature at a time and add it to the current feature set. Features are selected in a descending order of the discriminative power determined by the F-score. The feature added is the one that gives the highest accuracy.

Based on the properties that might be relevant to consensus shape prediction, we construct 17 features (the features are listed at our website) and compute the F-score for each of them. The accuracy is evaluated using a linear SVM method. The standard grid search approach is used to find an optimal SVM parameter. The performance of a feature set is evaluated using 5-fold cross validation. Prediction accuracy is the average value of all cross validation sets. The feature set that achieves the highest accuracy includes the following four features.

• *F1: the contribution of sequences*. We capture the contribution of sequences using the number of sequences mapped to the shape. This feature reveals how the shape is shared among the homologous sequences. *F*1 = |*P.LX*|.

• *F2: the contribution of structures*. This feature represents the abundance of structures mapped to the shape. *F*2 = |*P.LS*|.

• *F3: the average free energy*. Energy model is commonly used to determine the stability of predicted structures. The basic idea behind this feature is that a stable shape is expected to be derived from a group of stable structures. $F3=\frac{\sum_{s\in P.LS}\Delta G\left(S\right)}{\left|P.LS\right|}$.

• *F4: the average of minimal free energy*. This feature is different from *F*3 in that it considers only the minimal free energy among all predicted structures of each sequence. $F4=\frac{\sum_{X\in P.LX}MFE\left(X\right)}{\left|P.LX\right|}$.

#### Shape ranking using a simple scoring function

Once the features are determined, they are used together with a trained linear SVM for shape labeling. Multiple shapes might be labeled as "true". In order to rank these candidate shapes for the final shape selection, we evaluate each candidate shape using a score named *sc*, which is proportional to the signed distance between the candidate shape to the classification hyperplane [32]. Specifically, *sc *= *w *· *x *+ *b*, where · denotes the dot product, *w *is the weight vector, and *x *is the instance vector. *w *is trained on the optimization function in the linear SVM. The larger |*w_j_*| is, the more important the *j*th feature is. This is restricted to *w *in a linear SVM model.

#### Time complexity of shape prediction

For *N *input sequences, there are S predicted structures. These structure can be grouped into *P' *shapes. As we use the de novo folding tools to output near-optimal structures within a given energy range (e.g. 5%), we found that *N*: *S*: *P' *≈ 1: 10: 1:375. Mapping structures to shapes takes *O*(*SL*), where *L *is the sequence length. As sorting shapes according to their features takes *P'log*(*P'*) and *P' *≤ 2*N *and *S *≤ 11*N*, the procedure of shape prediction has time complexity *O*(*NL *+ *NlogN*).

### Consensus structure prediction given a shape

Once we determine the shape, we will predict the structure in the shape class for the given homologous ncRNAs. Structures corresponding to the same shape can differ significantly in the details of the loop and stacking regions. A strategy is needed to choose the correct structure inside the shape class for each input sequence. The simplest strategy is to output the MFE structure for the chosen shape, which has been used in previous work [12]. However, the MFE structure in a shape may not be the native structure. In particular, the accuracy of the MFE prediction for ncRNAs containing pseudoknots is low.

In this section we describe KnotStructure, a comparative structure prediction method for homologous sequences given the shape. The rationale behind comparative structure prediction is that the secondary structures and sequences are conserved during evolution. Thus, finding the structures to maximize both the sequence and the secondary structure similarity among homologous ncRNAs provides the basis for comparative structure prediction. Various methods for evaluating structural and sequence similarity exist. The major challenge is to efficiently select $\left|\hat{P}.LX\right|$ representative structures to achieve the highest structural and sequence similarity.

As we already derived the consensus shape $\hat{P}$ using KnotShape, only structures with shape $\hat{P}$ will be allowed. In addition, for each sequence $X_{i}\in \hat{P}.LX$, only one structure with shape $\hat{P}$ can be selected. The total number of combinations of structures for measuring the similarity is thus $\prod_{i=1\;to\;|\hat{P}.LX|}|\hat{P}.LS\cap S^{i}|$, where $\hat{P}.LS\cap S^{i}$ contains structures with shape $\hat{P}$ for a sequence *X_i_*. Although efficient heuristics exist to measure the similarity among multiple structures and sequences, the sheer amount of combinations poses a great computational challenge.

**Procedure 1 **Representative structures selection

**Input: **$\hat{P}$, $\hat{P}$.*LX*, $\hat{P}$.*LS*

**Output: **The representative structures

  1. Initialization

  **for **Every two structures $S_{i}^{x}$ and $S_{j}^{y}$**do**

    //only evaluate similarity of structures from different sequences

    **if ***x *≠ *y ***then**

      Evaluate the similarity of $S_{i}^{x}$ and $S_{j}^{y}$

    **else**

      $S_{i}^{x}$ and $S_{j}^{y}$ has similarity -∞

    **end if**

  **end for**

  2. Select the set of representative structures using hierarchical clustering

  //Each structure is a cluster by itself

  **repeat**

    Combine a pair of clusters with the highest similarity

    For any structure $S_{i}^{x}$ added to the cluster, remove all other structures $S_{j}^{x}$ for *j *≠ *i*

    Re-evaluate the similarity between clusters

  **until **the cluster has size $\left|\hat{P}.\text{LX}\right|$

In order to efficiently select representative structures, we use a similar method to Unweighted Pair Group Method with Arithmetic Mean (UPGMA), an agglomerative hierarchical clustering technique [33]. Each object (i.e. secondary structure) starts in its own cluster. The closest pair of clusters is selected and merged into a single cluster as one moves up the hierarchy. The distance between clusters is measured using arithmetic mean defined in UPGMA. Compared to the standard clustering procedure, we have constraints on the objects that can be selected into the same cluster. Given the shape, only structures that have shape $\hat{P}$ and come from different ncRNAs can be combined in the same cluster. The detailed clustering process is described in Procedure 1.

During clustering, the structural and sequence similarity is evaluated using grammar string-based approach [34,35]. Grammar strings encode both secondary structure and sequence information for an ncRNA sequence. Grammar string alignment score can accurately quantify the structural and sequence similarity of two ncRNAs. In addition, grammar string can encode pseudoknot structures [34,35]. For a sequence *X_i _*and one structure $S_{j}^{i}$ in the folding space of *X_i_, X_i _*and $S_{j}^{i}$ are encoded in a grammar string $gs_{j}^{i}$. We measure the similarity between any two grammar strings using the normalized grammar string-based alignment score over the alignment length. The similarity between groups of grammar strings is measured by arithmetic mean in UPGMA.

**Figure 4:**
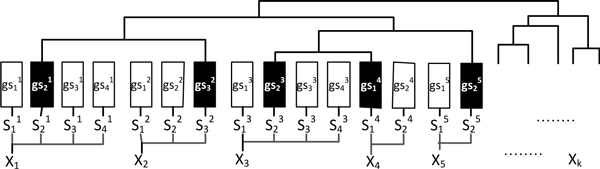
**An example of structure selection based on hierarchical clustering**. For each structure $S_{j}^{i}$ in the folding space of sequence *X_i_*, the grammar string encoding the structure and the sequence is denoted as $gs_{j}^{i}$. All sequences and their associated structures are converted into grammar strings before clustering. The highlighted rectangles indicate grammar strings that are selected as representative structures.

Figure 4 sketches the representative structure selection based on clustering procedure. Let $gs_{j}^{i}$ be a grammar string converted from *X_i _*and $S_{j}^{i}$, *X_i _*∈ *P.LX*. Once $gs_{j}^{i}$ is selected, all the other grammar strings derived from the folding space of *X_i _*will be removed from further processing.

The progressive MSA is performed on the set of representative structures using the clustering path as a guide tree. We then derive the consensus secondary structure from the alignment. The consensus structure can be mapped to each aligned sequence to accomplish the predicted structure of an individual sequence.

#### Running time of structure prediction

Converting a sequence and an associated secondary structure into a GS (grammar string) takes *O*(*L*^2^), where *L *is the length of the sequence. Let the number of structures in $\hat{P}.LS$ be *m*. It takes *O*(*L*^2^*m*) to encode all structures with shape $\hat{P}$. In the first step of hierarchical clustering, we measure the similarity between GSs of different ncRNAs by conducting all-against-all comparison. Conducting pairwise GS alignment takes *O*(*l*^2^), where *l *is the length of the GS sequence and *l *≤ *L*. By using the default energy cutoff (5%) for sub-optimal structure generation, we observed that *m *≤ 11*N*. Thus, the all-against-all similarity measure has time complexity *O*(*L*^2^*N*^2^). The guide tree generated using the clustering procedure contains at most *N *representative structures. Thus, the total running time for clustering is *O*(*L*^2^*N*^3^), which is the leading time complexity term for the consensus structure prediction algorithm.

## Experimental results

### Data sets

The training data set is the *K10 *from BraliBASE [36]. It contains 845 sequence sets, each of which has 10 homologous ncRNAs. There are two test data sets. The first one is the *K15 *from BraliBASE. *K15 *contains 503 sequence sets, each of which has 15 homologous ncRNAs. As existing shape prediction tools are not designed for handling pseudoknots, we use the pseudoknot-free sequence sets in *K15 *to compare the performance of shape prediction. After removing the sets containing pseudoknots, we have 452 sequence sets left. To test the performance of pseudoknot prediction, we constructed the second test set *R15 *from psuedoknot families of Rfam [3]. In Rfam 10.0, there are 71 families containing pseudoknots. 25 of them have published structures. Of the 25 families, only families with at least 15 seed sequences are used for testing our tools. For each chosen family, sets of 15 sequences are chosen randomly to construct the test sets. Finally *R15 *contains 160 test sets. The average pairwise sequence identities range from 60-93%. For all sequence sets, the reference shapes were derived from Rfam [3].

### SVM training

For both the training and testing data sets, we need to apply de novo folding tools to the sequences. We choose a folding tool using the following criteria. First, this tool is able to output both the optimal and sub-optimal structures. Second, this tool has high accuracy and can be efficiently applied to a large number of ncRNAs. Finally, if the target sequences contain pseudoknots, this tool should be able to output pseudoknot structures. As a result, we chose TT2NE [20]. Different from many other pseudoknot prediction tools that have constraints on the type of the pseudoknot, TT2NE is more exible about the types of the target sequences. However, when it was applied to *K10*, TT2NE failed to output structures for some sequences due to the length limit (200 nt) and also existence of IUPAC characters in some sequences. Thus, for the training data set *K10*, we applied quikfold [37] because *K10 *rarely contains pseudoknots. Although it is ideal to use the same folding tool to the training and testing data set to achieve optimal classification performance, the complexity of the training and test data sets together with the performance of de novo folding tools lead to the current combination. In the Discussion section we will briefly discuss how de novo folding tools affect the performance.

We employed the SVM model implemented by LIBSVM tool [38] for classification. For each sequence in *K10*, we applied quikfold with the energy range 5% to obtain both optimal and sub-optimal structures of each sequence. The predicted structures were grouped based on their corresponding shapes. Associated features were extracted and enclosed with each shape. We normalized feature values to fit the different properties of test sets to the same scale.

To label shapes, we used the shapes extracted from the consensus structures in Rfam [3] as the reference. Shapes are labeled according to their correctness. We label a shape as 1 if it is as same as the reference shape. Otherwise, it is labeled as -1.

### Shape prediction comparison

We compared KnotShape with RNAcast [12], which is part of RNAshapes package [25]. RNAcast takes the sequences as the input and predicts the consensus shape shared by all sequences. As it is not designed for pseudoknot structures, we only applied RNAcast to 452 test sets of *K15*, which are pseudoknot-free. TT2NE is applied to the test set using the default parameters. For each sequence, the optimal structure and 10 sub-optimal structures are kept as the sample of the folding space for each sequence. We compared our predicted shapes and the first-ranked shapes output by RNAcast with the reference shapes derived from Rfam [3]. The comparison is presented in Table 1. Note that RNAcast cannot output the shapes containing pseudoknots and thus is left blank for *R15 *in Table 1. The accuracy of KnotShape is 18% higher than RNAshapes.

**Table 1:** Accuracy of shape predictions

	*K15*			*R15*		
	Testset	Correct shapes	%Accuracy	Testset	Correct shapes	%Accuracy
KnotShape	452	311	68.81	160	107	66.88
RNAcast	452	232	51.33	-	-	-

### Structure prediction comparison

We applied the predicted shapes to pseudoknot structure prediction and compared the structure prediction performance with IPknot [19], HxMatch [39], and TurboKnot [21], which are chosen because of their popularity, availability, and easy usage on large number of sequences. Sequence alignments were generated using ClustalW and entered as the input to IPknot and HxMatch. For IPknot, we chose the appropriate levels of prediction according to the test sets. We ran Hxmatch with the default parameters. We used the parameters suggested in [21] to run TurboKnot. Sensitivity and the Positive Predicted Value (PPV) are used to evaluate the performance:

$$Sensitivity=\frac{TP}{TP+FN},\;PPV=\frac{TP}{TP+FP}$$

**Figure 5:**
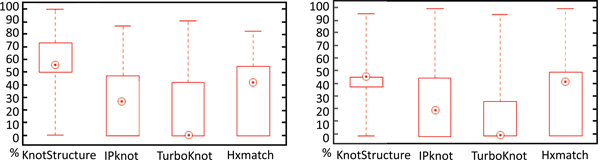
**Comparison of the sensitivity and PPV of different tools**.

*TP *is the number of correctly predicted base pairs. *FN *is the number of base pairs that are in the reference structure but not in the predicted structure. *FP *is the number of base pairs that are in the predicted structure but not in the reference structure. Figure 5 is the boxplot of the sensitivity and PPV over all 160 test sets. KnotStructure has the best overall performance on the whole data set. The median values of sensitivity and PPV are 54.55% and 46.15% for KnotStructure. Hxmatch has the next highest sensitivity and PPV (42.11% and 42.86% respectively). The abstract shapes of these families are shown in Table 2. Three families contain simple H-type pseudoknots while the other three families contain more complicated pseudoknots. In order to show the effect of shape prediction in structure prediction, we predicted the structures of *R15 *using 10 randomly selected shapes. The average sensitivity and PPV of predicted structures with the predicted shapes are higher than those using random shapes as shown in Table 3. Table 4 shows the average sensitivity and PPV over all sequences of each family compared to other tools. The average running time of KnotStructure on each family compared to other tools is shown in Table 5.

**Table 2:** Abstract shapes of ncRNA families in R15

RNA Type	Shape Level 5	Shape Level 3	RNA Type	Shape Level 5	Shape Level 3
HDV _ribozyme	[A[B]]b[]a	[AA[B]]b[[[[[[]]]]]]aa	Alpha_RBS	[ABC]bac	[[[ABC]]]bac
Tombus_3_IV	[[]A][][]a	[[[]A]][][]a	Tymo_tRNA-like	[][][]A[a]	[][][[]]AA[aa]
Corona_FSE	[A]a	[AA]aa	Prion_pknot	[A]a	[A]a

**Table 3:** Sensitivity and PPV of predicted structures using the predicted shapes and randomly selected shapes

	Predicted shape	Randomly selected shape									
		1	2	3	4	5	6	7	8	9	10
SEN	79.00	45.68	58.41	53.09	58.06	55.74	45.88	44.75	56.12	61.08	46.54
PPV	67.10	38.81	50.92	42.82	49.15	46.24	40.29	36.33	45.75	52.80	38.53

**Table 4:** Sensitivity and PPV for different ncRNA families

			Sensitivity				PPV			
RNA Type	Len^⊕^	TS*	Knot-Structure	IP-knot	Turbo-Knot	Hx-match	Knot-Structure	IP-knot	Turbo-Knot	Hx-match
HDV_ribozyme	89.70	12	**82.52**	50.66	36.47	23.51	**80.13**	59.24	39.28	39.77
Alpha_RBS	110.99	18	**74.36**	46.59	46.24	24.49	**40.59**	25.34	23.70	22.19
Tombus_3_IV	91.61	4	**84.00**	65.91	72.00	80.00	83.14	78.02	73.47	**90.91**
Tymo_tRNA-like	85.12	3	**96.79**	76.41	83.52	75.09	**90.25**	73.62	86.85	90.11
Corona_FSE	82.91	9	**96.14**	56.55	56.55	73.27	75.65	60.51	42.19	**92.30**
Prion_pknot	40.46	114	**40.17**	13.70	2.96	30.26	**32.85**	15.71	3.03	28.86

Bold number and underlined number indicate the highest and the second highest sensitivity or PPV for each family. ^⊕ ^Average sequence length. * The number of test sets.

**Table 5:** Running time for different ncRNA families (seconds)

RNA Type	Knot-Structure	IP-knot	Turbo-Knot	Hx-match	RNA Type	Knot-Structure	IP-knot	Turbo-Knot	Hx-match
HDV_ribozyme	1.55	0.35	28.22	0.41	Tymo_tRNA-like	1.78	0.24	12.24	0.28
Alpha_RBS	1.93	0.49	37.25	0.55	Corona_FSE	1.51	0.30	12.75	0.35
Tombus_3_IV	1.80	0.37	13.66	0.42	Prion_pknot	1.07	0.10	4.23	0.12

## Discussion and conclusion

Based on the fold-then-align strategy, choice of folding tools can play an important role in the performance of the shape and structure prediction. For the test set, we tested two folding tools: HotKnots [40] and TT2NE. We used them in three different ways: Hotknots, TT2NE, and both of them. We ran HotKnots and TT2NE with default parameters. The experimental results show that using TT2NE alone achieves the best performance in consensus structure prediction. It is likely that other folding tools exist to yield better performance than TT2NE. However, as the performance of those tools also depends on the input data and the parameters, a systematic study is needed to choose the best tool.

For TT2NE, we currently only use 10 sub-optimal structures. Increasing this number moderately does not affect the performance significantly. It indicates that the correct structures have high rankings in the folding space. However, there are a few sequence sets for which the correct structures are not near-optimal. Thus, enlarging the sample folding space will likely increase the sensitivity. However, using a large number of sub-optimal structures can increase the computational cost. Thus, a better algorithm is needed to achieve a better tradeoff between sensitivity and running time. This is an important part of our future work.

There are more pseudoknot-free structures available than pseudoknot-containing structures. To achieve a reliable SVM model, more training data is desired. We used *K10 *for feature selection. This may cause KnotShape to have slightly lower predictive performance on pseudoknot-containing than pseudoknot-free sequences. Nonetheless, the features used in KnotShape does not heavily rely on the free energy value, which is different between pseudoknot-free and pseudoknot-containing structures. Instead, the feature set is based on multiple RNA properties shared among homologous sequences.

Extensive analysis of RNA properties based on SVM allows us to identify important features related to abstract shapes. The combination of mass data analysis and SVM-based feature ranking makes KnotShape a promising tool for shape prediction. By combining the predicted shapes and the multiple structural alignment strategy, KnotStructure demonstrates higher accuracy in pseudoknot structure prediction.

## Competing interests

The authors declare that there are no competing interests.

## Authors' contributions

YS initiated the project and participated in its design and coordination. RA designed the shape prediction algorithm, developed both KnotShape and KnotStructure, and conducted the experiments. RA and YS are both responsible for writing the manuscript.

## Declarations

The publication costs for this article were funded by NSF DBI-0953738 and IOS-1126998.

This article has been published as part of *BMC Bioinformatics *Volume 14 Supplement 2, 2013: Selected articles from the Eleventh Asia Pacific Bioinformatics Conference (APBC 2013): Bioinformatics. The full contents of the supplement are available online at http://www.biomedcentral.com/bmcbioinformatics/supplements/14/S2.
